# Lessons to cancer from studies of leukemia and hematopoiesis

**DOI:** 10.3389/fcell.2022.993915

**Published:** 2022-09-20

**Authors:** Geoffrey Brown

**Affiliations:** School of Biomedical Sciences, Institute of Clinical Sciences, College of Medical and Dental Sciences, University of Birmingham, Birmingham, United Kingdom

**Keywords:** leukemia, leukemia stem cells, cancer stem cells, oncogenes, therapies

## Abstract

The starting point to describing the origin and nature of any cancer must be knowledge about how the normal counterpart tissue develops. New principles to the nature of hematopoietic stem cells have arisen in recent years. In particular, hematopoietic stem cells can “choose” a cell lineage directly from a spectrum of the end-cell options, and are, therefore, a heterogeneous population of lineage affiliated/biased cells. These cells remain versatile because the developmental trajectories of hematopoietic stem and progenitor cells are broad. From studies of human acute myeloid leukemia, leukemia is also a hierarchy of maturing or partially maturing cells that are sustained by leukemia stem cells at the apex. This cellular hierarchy model has been extended to a wide variety of human solid tumors, by the identification of cancer stem cells, and is termed the cancer stem cell model. At least, two genomic insults are needed for cancer, as seen from studies of human childhood acute lymphoblastic leukemia. There are signature mutations for some leukemia’s and some relate to a transcription factor that guides the cell lineage of developing hematopoietic stem/progenitor cells. Similarly, some oncogenes restrict the fate of leukemia stem cells and their offspring to a single maturation pathway. In this case, a loss of intrinsic stem cell versatility seems to be a property of leukemia stem cells. To provide more effective cures for leukemia, there is the need to find ways to eliminate leukemia stem cells.

## Introduction

There are very many different types of leukemia. Since the late 19th century, their diagnosis and subtyping were underpinned by the leukemia cells’ appearance, including their relationship to a cell lineage, maturation status, and the rapidity of proliferation. Based on these features, the four major subtypes of leukemia are chronic lymphocytic leukemia (CLL), chronic myeloid leukemia (CML), acute lymphoblastic leukemia (ALL), and acute myeloid leukemia (AML). The acute leukemias were subdivided from examination of a combination of morphologic, immunotypic, and cytogenetic features. For example, the French-American-British classification of AML recognized eight different subtypes, designated as M0 through to M7. In 2008, the WHO updated the classification of the malignancies of hematopoietic and lymphoid tissues incorporating layer upon layer of complexity, including morphogenic, immunotypic, cytogenetic, and molecular genetics findings. The WHO guide to clinicians listed 94 disease entities (Swerdlow et al., 2008).

A precise diagram for how each blood cell type develops from HSCs is central to resolving how this is distorted in the different leukemias. Hematopoietic stem cells (HSCs) sit at the apex of blood cell development, and for over 30 years they were regarded as a uniform cohort of cells that is uniquely able to self-replicate indefinitely. In classic models, HSC developmental progression is depicted in a branching tree-like manner (Spangrude et al., 1988). Lineage options are restricted stepwise *via* a series of binary decisions and compartmentalized intermediate hematopoietic progenitor cells (HPCs) that ultimately give rise to lineage-restricted HPCs. These cells have a lesser ability to self-renew. In addition to undergoing expansion, lineage-restricted HPCs differentiate further to give rise to mature blood cells that are functional and predominantly short-lived. (Kondo et al., 2003).

From around 2009, new principles to the nature of HSCs arose leading to continuum (Ceredig et al., 2009) and diffusion map (Nestorowa et al., 2016) models for hematopoiesis. In these models, HSCs “choose” a cell lineage directly from a spectrum of end-cell options; they are, therefore, a consortium of HSC subtypes with distinct characteristics. Known subsets include HSCs that are megakaryocyte (Sanjuan-Pla et al., 2013), lymphoid-, myeloid- and dendritic cell lineage-biased (Challen et al., 2010; Gekas and Graf, 2013; Shimazu et al., 2012; Naik et al., 2013), and erythroid and macrophage affiliated (Mooney et al., 2017). The identification of these lineage-biased/affiliated cells is important because they, like multipotent HSCs, are primarily at risk of leukemogenesis by virtue of their ability to self-renew. HPCs normally undergo a limited number of cell divisions. There are not findings to support the view that an oncogene can endow a committed HPC with the capacity to self-renew which is required to sustain leukemia. In continuum and diffusion map models, lineage imprinting occurs earlier than previously thought, and the differentiation of HSCs and their downstream HPCs is a progressive process with broad developmental trajectories (Nestorowa et al., 2016). This allows cells to move to the left or right of a chosen pathway (Ceredig et al., 2009; Nestorowa et al., 2016); in other words, HSCs and HPCs having chosen a pathway remain versatile (Brown and Ceredig, 2019). Therefore, an important consideration in the transformation of multipotent HSCs is whether the transformed cell remains versatile to generate leukemia cells belonging to several cell lineages.

In this review, I focus on how a clearer picture of the involvement of HSCs in leukemogenesis has emerged from advances in our understanding of hematopoiesis, the influence of oncogenes on HSCs, and that the human leukemias are sustained by leukemia stem cells (LSCs) at their apex. In this regard, the leukemic cell hierarchy is similar to that of normal hematopoiesis. However, unlike normal stem cells, the progeny of LSCs seems to be restricted to a cell lineage. The principle of LSCs extends to solid cancers from the identification of cancer stem cells (CSCs), and consideration is given to finding ways to eliminate these cells.

## The stem cell origin of some leukemias

CML and some AMLs originate in an HSC. In 1978, the expression of the glucose-6-phosphate dehydrogenase (G-6-PD) isoenzymes A and B were examined for cells from three women with CML. From X chromosome inactivation, only one *G-6-PD* gene is active in each female cell which is a mixture of cells that express either the A or B isoenzyme. One enzyme type was found in the blood granulocytes, erythrocytes, platelets, monocytes/macrophages, and B lymphocytes of the patients. The investigators concluded that the clonal origin of CML is a multipotent HSC, and coined the term LSC. Their findings also revealed that the above blood cell types arise from a common HSC (Fialkow et al., 1978). For the subtypes of AML analyzed in 1997, the investigators concluded that they had arisen from a primitive hematopoietic cell. The cells that initiated human AML in non-obese diabetic-severe combined immunodeficient (NOD/SCID) mice were CD34^++^, CD38^−^ and able to self-renew, which are attributes of a normal HSC (Bonnet and Dick, 1997).

The advent of panels of monoclonal antibodies facilitated and strengthened the sub-typing of the leukemias regarding their affiliation to a cell lineage. Some leukemias were assigned as arising in a committed HPC from the well-described phenotypes of normal HPC sub-populations. However, further analyses of leukemias that were thought to originate in a committed HPC have revealed a more primitive origin. In acute promyelocytic leukemia (APL), there is a differentiation block at a promyeloid stage of myeloid differentiation, leading to an abundance of promyelocytic blasts. The hallmark of APL is the chromosomal translocation t (Edwards et al., 1999; Adolfsson et al., 2005) which gives rise to the fusion protein PML/RARα. The presence of translocation and fusion protein has not been reported in any other malignancy, and the protein plays a crucial role in APL leukemogenesis. The translocation and oncoprotein were observed to be present in CD34^+^ CD38^−^ HSC-like cells from patients with APL, supporting a disease origin in an HSC rather than a committed myeloid HPC (Edwards et al., 1999). Pro-B cells accumulate in the bone marrow of infant t (Ceredig et al., 2009; Mooney et al., 2017) *MLL-AT4* B-cell precursor ALL. A new view is that the cell of origin is a fetal liver lymphoid-primed multipotent progenitor (LMPP) (Malouf and Ottersbach, 2018). These stem-like cells generate neutrophils, monocytes, B cells, and T cells, but do not produce significant erythroid cells and megakaryocytes (Adolfsson et al., 2005). The cells in childhood B-ALL express the early stage B-cell antigen CD19 and that a B-cell committed progenitor is the cell of origin is a longstanding notion (Greaves, 1981). There are findings to support a more primitive HSC-like cell as the cell of origin. For children with B-ALL, cytogenetically aberrant cells are present in the CD34^+^, CD33^−^, CD38^−^, CD19^−^ primitive cell compartment in the bone marrow (BM), and engraftment of B-ALL into NOD/SCID mice was achieved with CD34^+^, CD10^−^ cells or CD34^+^, CD19^−^ cells (Quijano et al., 1997; Cox and Blair, 2005; Hirt et al., 2011). A surprising finding was that transformation of a self-renewing HSC gives rise to CLL, as opposed to the CLL subtypes arising from the transformation of an antigen-experienced B-cell (Alizadeh and Majeti, 2011; Kikushige et al., 2011). A striking feature of the above LSCs is that they, unlike normal HSCs, have given rise to offspring belonging to just one cell lineage. There is excessive production of neutrophils in CML, promyelocytic blasts predominate in APL, and B-cell lineage cells prevail in infant B-cell precursor ALL, childhood ALL, and CLL.

## The cancer stem cell model

The CSC model of cancer captures the importance of stem cells to the genesis of cancer. The proposal arose from studies of AML and, as mentioned above, the NOD/SCID leukemia- initiating cells were rare, on average 1 per 10^6^ (Dick, 2008). These are LSCs that sustain a hierarchy of maturing or partially maturing leukemia cells. This cellular hierarchy model was extended to solid cancers, and universality is important because CSCs are also largely responsible for disease relapse and metastasis. Studies of different human solid cancers have led to the conclusion that the cells that give rise to solid tumors when transplanted into NOD/SCID mice are also often quite rare (0.1%–0.0001%). For breast cancer, CSCs are a small fraction of tumor cells and are viewed as seeding the tumor. The population of breast cancer CSCs is heterogeneous; the two subgroups, namely a mesenchymal and quiescent type and an epithelial and proliferative type, are two dynamic states of breast cancer CSCs (Zhou et al., 2019). For colorectal cancer, CSCs are central to tumor initiation because when isolated from primary tumors they give rise to tumors in mice. The development of a better understanding of biomarkers is assisting the clarification of colorectal CSC phenotypes (Munro et al., 2018). CSCs have been identified and isolated for other solid cancers, including bladder (She et al., 2008), head and neck squamous cell carcinoma (Prince et al., 2007), lung (Eramo et al., 2008), pancreatic (Hermann et al., 2007), prostate (Collins et al., 2005), and sarcoma (Wu et al., 2007). For these diseases, CSCs play a role in disease initiation and metastasis.

A low frequency of CSCs is not always the case because around 27% of unselected melanoma cells from four patients gave rise to a tumor in single cell transplants when a modified xenotransplantation assay was used. The investigators concluded that melanoma follows the principle of the cancer stem cell model, and that CSCs are common in some tumors (Quintana et al., 2008). For some solid tumors there is still a lack of information about the frequency of CSCs. For example, glioblastoma is the most malignant primary brain tumor. It contains CSCs that have the ability to generate neurospheres and that are viewed as contributing to tumor initiation and therapeutic resistance. The frequency of CSCs within glioblastoma is uncertain because the linkage of markers between normal stem cells and CSCs is still controversial (Lathia et al., 2015).

## At least two “hits” are needed for leukemia

Studies in 1983 showed that at least two genomic insults are needed to transform primary embryo fibroblasts so that these cells give rise to large tumors in mice. Cooperating oncogenes included, for example, activation of a *RAS*-like and a *MYC*-like gene. (Land et al., 1983). Of importance was whether the same is true for cancer in humans, and, at least, two insults are needed as seen for the genesis of leukemia in children.

Twins with concordant leukemia provided a unique opportunity to unravel the etiology of leukemia’s. Embryos that split after day 3 and before day 7 develop as separate embryos with a single (monochromic) placenta. This is the case for 60% of monozygotic twins and there is blood cell chimerism, via vascular anastomosis. Some years ago, it was suggested that concordant leukemia in twins has a common, and often prenatal, origin. In this case, leukemia is initiated in one of the twins, and the progeny of the clone transfers to the other twin *via* a shared placental (Wolman, 1962). A number of studies of concordant leukemia have provided evidence to support the sharing of a leukemia initiating clone from one twin to the other (reviewed in 37) An identical DNA rearrangement to the immunoglobulin heavy chain was seen to be shared by B-cell lineage ALL cells from a pair of infant Siamese twin boys who were separated 42 days after birth and both diagnosed with ALL 7 months later. The investigators concluded for twins diagnosed in the first 1–2 years of life with leukemia that a single neoplastic transformation had occurred *in utero*, rather than there was a strong genetic susceptibility (Pombode de Oliveira et al., 1986). Cytogenetic studies in 1998 showed that the leukemia cells from pediatric monozygotic twins with concordant AML had identical clones by virtue of the shared karyotype +8, inv16, and +21. The twins developed AML at 3 and 4 years. At the time of the bone marrow transplant of the first twin, the abnormal clone was seen to be present in the asymptomatic twin. This finding again pointed to *in utero* twin-twin transfusion, and that a pre-leukemic clone had evolved years before the onset of overt disease. The investigators proposed that information about an acquired genetic change(s) in one twin may be useful to assessing disease in an asymptomatic twin (Richkind et al., 1998).

Molecular analyses of twins with concordant leukemia have substantiated sharing of a leukemic clone from one twin to the other. Eighty percent of cases of infant ALL (<1 year of age) have a reciprocal chromosome translocation involving the *MLL* gene (at 11q23). The *MLL* gene fuses principally with the *AF4* gene, and various other genes. Southern blots of *MLL*, with multiple enzyme digests, revealed that the ALL cells from three pairs of infant identical twins had identical restriction fragments. The restriction fragments from the DNA of a pair of twins co-migrated, and the migration patterns were different between the pairs of twins and for the controls. This study also showed that the *MLL* rearrangement had occurred *in utero* (Ford et al., 1993). Twenty-five percent of patients with childhood B-cell precursor ALL have the chromosome translocation t (12; 21) resulting in the *TEL(ETV6)-AML1(RUNX1)* fusion gene. ALL cells from twins with concordant leukemia were characterized by cloning (Ford et al., 1998) or the use of the polymerase chain method and sequencing (Wiemels et al., 1999a; Wiemels et al., 1999b; Maia et al., 2001). The latter method was used to track leukemogenesis in a triplets pregnancy, and two of the three triplets developed ALL (Maia et al., 2001). The same *TEL* and *AML1* breakpoints were observed to be present within the ALL cells for each of four pairs of twins.

There was often a latency to the development of *TEL-AML1* ALL in one of the twins. In one study, monozygotic twins were diagnosed with *TEL-AML1* ALL at 3 years, 6 months and 4 years, 10 months (Ford et al., 1998). The latency can be very protracted as in a different study the diagnoses of *TEL-AML1* ALL were at ages 5 and 14 (Wiemels et al., 1999a). Examination of an archived slide of BM cells from the twin who developed leukemia later showed that the *TEL-AML1* fusion sequence was present in the archived marrow. At the time, the marrow was haematologically normal. The *TEL-AML* fusion gene rearrangement has, therefore, a fetal origin. Moreover, the most plausible explanation for the above findings is that a leukemia-initiating mutation had occurred *in utero*, this clone was shared between the children, and a second, or more, insult(s) was needed for full-blown childhood ALL (Ford et al., 1998). Evidence from epidemiological studies of older children with *TEL-AML1*–positive or hyper-diploid ALL favours an abnormal response to infection playing a causal role regarding the second insult (Greaves et al., 2003). The rationale is that the naïve immune network is modulated by exposure to common infections, and a lack of exposure in the first year of life leads to a risk of leukemia by virtue of a dysregulated immune response. It may then be possible to develop a prophylactic to prevent ALL in children.

## The pre-leukemia stem cells arising from hematopoietic stem cells

HSCs may give rise to many leukemias and the two-hit model of leukemogenesis states that an HSC with a stable and pre-leukemia lesion must only progress to full-blown leukemia after a second hit. A pre-LSC clone having received just one insult is likely, therefore, to be able to undergo normal hematopoiesis. There is good evidence to support this view. Five percent of healthy newborns have a *TEL-AML1* fusion, as shown by DNA-based GIPFEL screening. This is compatible with normal hematopoietic development in the majority of the cases because ninety-nine percent of newborns will never develop B-cell precursor ALL (Schafer et al., 2018). Therefore, the TEL-AML1 transcription factor has a very low oncogenic potential, which strengthens the importance of the need for a second hit that is either spontaneous or environmental.

As mentioned above, blood granulocytes, erythrocytes, platelets, monocytes/macrophages, and B lymphocytes are all part of the *BCR-ABLp210+* pre-LSC and HSC-derived CML clone with pre-LSCs differentiating into mature blood cells. AML1–ETO fusion transcripts are detectable in most patients with t (8; 21) AML, and were also found in various colony-forming cells, including mixed (CFU-Mix), erythroid (BFU-E), granulocyte-macrophage (CFU-GM), and megakaryocyte (CFU-Meg), when BM cells were examined for patients who had been in remission for 12–150 months (Miyamoto et al., 1996). Later studies showed that transcripts were detectable within HSCs, monocytes, and a fraction of B cells, but not T cells, and again in up to 60% of CFU-Mix BFU-E, CFU-GM, and CFU-Meg. The morphologies and compositions of the AML1-ETO1+ and AML1-ETO − myeloid colonies were the same. (Miyamoto et al., 2000). Hence, the t(8; 21) acquisition had occurred at the level of an HSC, and these cells can differentiate into various myeloid and B-cells. The investigators concluded that *AML1–ETO* is an early step in the process that leads to transformation and that a further insult might involve the machinery for myeloid lineage development.

## Signature mutations in leukemia

As considered above, analyses of genetic abnormalities have greatly assisted the sub-typing of leukemias. Moreover, whole genome sequencing has now transformed our understanding of the various types and the pathogenesis of hematopoietic malignancies. For example, the Pediatric Cancer Genome Project looked to sequence 600 pediatric cancers and matched germline cancers (Downing et al., 2012). Aims were to fully explore the mutations that drive cancer and their frequency within specific cancers. As to frequency and from earlier studies of leukemias, some types of leukemia are linked to a specific oncogenic event (Table 1). The overt leukemia cells closely resemble a normal differentiated or partially differentiated cell of a particular lineage. One interpretation of these findings is that the signature mutation plays a role in specifying the lineage fate of the LSCs and their offspring.

**TABLE 1:** Signature mutations in leukemia.

Leukemia	Oncogenic event	Frequency
Lymphoid		
Infant B-cell ALL	*MLL-AF4* fusion gene	80%
Childhood B-cell ALL	*TEL-AML1* fusion gene	25%
Adult B-cell ALL	*PAX5* mutations	34%
T-cell leukemia	*LMO2* gene activation	Typifies
Extranodal MALT lymphomas	*MLT/MALT1* involvement	26%
Myeloid		
APL	*PML-RARA* fusion gene	Typifies
M1, M2, and M4 AML	*CEBPA* mutations	7%–20%
Adult AML	*NPM1* mutations	25%–41%
t (8; 21) AML	*AML1–ETO* fusion gene	Typifies
CML	*BCR-ABLp210* oncoprotein	Typifies
Ph+ precursor B-cell ALL	*BCR-ABLp190* oncoprotein	Typifies

Some of the genes that are involved in the genetic abnormalities shown in Table 1 encode transcription factors (TFs) that are important to HSC development towards an end cell type. The *PAX5* gene is frequently mutated in human B-cell leukemia, and encodes a transcription factor that regulates the early stages of B-cell development (Fuxa and Skok, 2007). The *LM02* gene encodes a TF that is activated by chromosomal translocations in T-cell leukemia. It is expressed within immature CD4/CD8 double negative thymocytes but does not have an obligatory role in T-cell development, as revealed by studies of knockout mice (McCromak et al., 2003). However, the LMO2 TF may still have a regulatory role. The *PML-RARA* fusion gene typifies APL and the RARA gene encodes the TF retinoic acid receptor (RAR) α. Like LMO2, RARα does not have an obligatory role in myelopoiesis but is a key regulator of the differentiation of granulocyte-macrophage progenitor cells (Kastner and Chan, 2001). *CEBPA* is mutated in M1, M2, and M4 AML, and encodes the TF C/EBPα which is a regulatory switch that induces granulocyte lineage commitment in CFU-GM (Radomska et al., 1998).

The rest of the genetic abnormalities shown in Table 1 have an indirect influence on gene transcription. The main player in mucosa-associated lymphoid tissue (MALT) lymphomas is MALT1. MALTI is a paracaspase and a complex of BCL10, CAMA1, and MALT1 activates the transcription factor NF-κB, *via* IKKγ degradation (Bertoni and Zucca, 2006). The AML1-ETO protein is viewed as regulating gene expression *via* epigenetic mechanisms. The fusion protein recruits multiple chromatin-modifying enzymes to target genes, to alter chromatin marks and transcription factor binding (Rejeski et al., 2021). The *MLL1* gene that is rearranged in infant B-cell ALL encodes the histone lysine-specific methyltransferase 2A which plays a role in gene expression (Winters and Bernt, 2017). Mutation of nucleophosmin (NPM1) is a common driver in adult AML. NPM1 is present in a small region inside the nucleus and is a multifunctional protein that has roles in the maintenance of genomic stability and chromatin remodeling, the biogenesis of ribosomes, the p53-dependent stress response, and the modulation of pathways that suppress cell growth (Box et al., 2016; Heath et al., 2017).

## Oncogene-mediated setting of hematopoietic stem cell fate

In keeping with the link between signature mutations and TFs that play a role during hematopoietic cell development along a pathway, some oncogenes can set the cell lineage of HSCs/HPCs and their offspring. This was revealed by targeting the expression of particular oncogenes to HSCs/HPCs in transgenic mice (Table 2).

**TABLE 2:** Targeting of oncogenic events in transgenic mice and the leukemia outcomes.

Oncogenic event(s)	Targeted cells	Transgenic mice outcome
*LMO2* gene activation	HSCs/HPCs, B-cells	T-cell ALL
*BCR-ABLp210* fusion	HSCs/HPCs	CML
*BCR-ABLp190* fusion	HSCs/HPCs	Precursor B-cell ALL at 13%
*BCR-ABLp190* & PAX5 loss	HSCs/HPCs	Precursor B-cell ALL at 90%
*TEL/AML1* fusion	HSCs/HPCs	B- and T-cell leukemias
*TEL/AML1* fusion & KDM5C loss	HSCs/HPCs	B-ALL at 22%

As mentioned above, *LMO2* is expressed within immature thymocytes in the thymus, with aberrant expression in these cells leading to T-ALL (Chambers and Rabbitts, 2015). LMO2 expression was initiated and maintained in only HSCs/HPCs by placing expression under the control of the stem cells antigen-1 (Sca1) promoter. These mice developed an aggressive T-cell ALL. Within HSCs/HPCs, the *LM O 2* oncogene, therefore, imposes aggressive T-cell ALL. T-cell ALL also developed when LMO2 expression was targeted, *via* appropriate Cre-mediated activation, to pro-B and germinal center B-cells. *LMO2* oncogene expression had, therefore, reprogrammed the B-cell lineage cells to favor the initiation of T-cell ALL and/or imposed the characteristics of T-cell ALL (Garcia-Ramirez et al., 2018). LMO2, when transfected with five other TFs (RUNX1T1, HLF, PRDM5, PBX1, and ZFP37), has also been shown to impart multilineage potential onto committed myeloid and lymphoid HPCs and myeloid effector cells (Riddell et al., 2014). To allow the LMO2 reprogramming of B-cell lineage, and other cells, to take place the cells must be inherently plastic. This is in keeping with the above view on the versatility of normal HSCs/HPCs.

*BCR-ABLp210* was targeted to HSCs/HPCs in transgenic mice via *Sca1-BCR-ABLp210*. The mice developed leukemia resembling human CML (Perez-Caro et al., 2009), revealing that this oncogene alone can impose CML within HSCs/HPCs. In this case, *BCR-ABLp210* seems to be less reliant on a cooperating secondary hit. DNA methylation at CpG islands was examined for HSCs/HPCs from the *Sca1-BCR-ABLp210* transgenic and the wild-type mice as to whether there had been a change to the epigenetic landscape. DNA hypomethylation was observed for the HSCs/HPCs from the *Sca1-BCR-ABLp210* mice. This was a lasting change because hypomethylation was also observed for the mature leukemic myeloid cells from the *Sca1-BCR-ABLp210* mice, despite an absence of oncogene expression in the mature cells. DNA methyltransferase 1 (Dnmt1) was upregulated within HSCs/HPCs from the *Sca1-BCR-ABLp210* mice. Expression of *Dnmt1* in HSCs/HPCs, under control of the endogenous *Sca1* promoter, led to DNA hypomethylation that was similar to that observed for the cells from the *Sca1-BCR-ABLp210* mice. The *Sca1-Dnmt1* transgenic mice developed malignancies that were mostly myeloid with a marked expansion of granulocytes in the bone marrow and blood. The investigators concluded that *BCR-ABLp210* had reprogrammed the epigenome of HSCs/HPCs to lead to CML (Vicente-Duenas et al., 2019).

In contrast to the influence of *BCR-ABLp210*, targeting of *BCR-ABLp190* to HSCs/HPCs in transgenic mice, *via Sca1-BCR-ABLp190*, led to precursor B-cell ALL resembling the human disease. In an aging mouse colony, the penetrance was only 13%. In transgenic mice that were double *Sca1-BCR-ABLp190* and *Pax5* ^*+/−*^ (a B-cell TF), precursor B-cell ALL developed with much shorter latency and a 90% incidence, and there were alterations in the remaining wild-type *Pax5* allele. Therefore, *BCR-ABLp190* together with the loss of *Pax5* can confer the changes that are needed for precursor B-cell ALL, whereby *Sca1-BCR-ABLp190* has set the cell lineage of HSCs/HPCs. Regarding the role of Pax5, metabolic genes were upregulated and glucose uptake and energy metabolism were increased within the pro-B cells from the *Pax5* deficient mice (Martin-Lorenzo et al., 2018).

As considered above, the *TEL/AML1* fusion gene confers a low risk of developing childhood B-ALL, and an abnormal reaction to infection has been postulated to trigger the overt disease. Transgenic mice failed to develop B-ALL when *TEL/AML1* expression was targeted to pro-B cells and the mice were exposed to natural infections. T-ALL and B-ALL were triggered when *TEL/AML1* expression was initiated in HSCs/HPCs and the mice exposed to infections. The H3K4me3 and H3K4me2 demethylase KDM5C is missense mutated in mouse *TEL/AML1* B-ALL and human relapse *TEL/AML1* B-ALL (Rodriguez-Hernandez et al., 2017). For mice that expressed *TEL/AML1,* introducing a loss of *KDM5C* into the B-cell compartment did not give rise to B-ALL. By contrast, loss of KDM5C in HSCs/HPCs that expressed *TEL/AML1* led to the development of B-ALL in mice (22%) that were kept in a special pathogen-free environment. Therefore, *TEL/AML1* can trigger T-cell and B-cell leukemias, and the second “hit” determines the lineage fate of *TEL/AML1*+ leukemia, with both “hits” having to occur in an HSC/early HPC (Rodriguez-Hernandez et al., 2021).

Different mutant RUNX1 oncoproteins have been shown to program alternative trajectories for hematopoietic cell differentiation. Four types of RUNX1 oncoproteins were assessed by their induced expression in embryonic stem cells. RUNX1-ETO reduced the expression and binding of PU.1 and C/EBPα (both TFs regulate myeloid differentiation) and led to a bias towards a B cell identity. Expression of R2010, which has a mutation in the DNA binding domain, reduced the interaction of wild-type RUNX1 with CBFβ (a master regulator of hematopoiesis) and increased GATA1 binding (a TF for erythropoiesis), leading to a bias away from megakaryocyte differentiation. RUNX1-EV11 reduced differentiation towards myeloid and erythroid cells (Kellaway et al., 2021). RUNX1 plays a role in the organization of the chromatin landscape for hematopoiesis (Lichtinger et al., 2012), and expression of the mutant proteins perturbed chromatin priming of lineage-specific sites, in addition to the regulatory TF network (Kellaway et al., 2021).

For the leukemias in the transgenic mice, LSCs appear to have been programmed by the “hit” to adopt a particular maturation pathway. For *BCR-ABLp210* and *RUNX1*, investigators have shown that there was disruption to the epigenome/chromatin priming within stem cells, and the demethylase KDM5C is missense mutated in human relapse *TEL/AML1* B-ALL. Dysregulation of epigenetics is viewed as an overarching hallmark of cancer. Therefore, it is perhaps not too surprising that a “hit” dysregulates the epigenetics to cell lineage commitment. A second “hit” might include lesions in genes such as *MYC* and *RAS* that highjack the control on cell proliferation leading to hyper-proliferation, and/or in genes that, for example, lead to loss of p53 function and BCL2 overexpression leading to inhibition of apoptosis. These lesions are forces in driving diverse human cancers and, for example, *MYC* and *RAS* are viral oncogenes within the highly oncogenic retroviruses.

## Conventional therapies spare leukemia stem cells

Chemotherapy and radiotherapy target cells that are cycling and are often very effective against the bulk of cancer cells. An important finding, in 1999, was that when quiescent LSCs were isolated from patients with CML they were found to be insensitive to high doses of chemotherapy (Holyoake et al., 1999). Second-generation drugs, e.g., dasatinib, nilotinib, and bosutinib, that inhibit the tyrosine activity of the BCR-ABLp220 protein are the main line treatment for CML and are used also to maintain remission. CML-LSCs are resistant to tyrosine kinase inhibitors, despite the expression of BCR-ABLp220 protein (Graham et al., 2002; Corbin et al., 2011). Tyrosine kinase inhibitors are also used to treat other cancers, including breast, kidney, liver, lung, pancreatic, prostate, and thyroid. They have been heralded as a breakthrough targeted therapy, as to having also a low toxicity profile even in older patients. Even so, the effectiveness of tyrosine kinase inhibition has been limited by the emergence of resistant clones (Pottier et al., 2020), and the second-generation tyrosine kinase inhibitors have only partially overcome the resistance conferred by the T3151 mutation.

It becoming clear that CSCs are resistant to conventional therapies. For example, the most common cancer globally is lung cancer and lung CSCs, which are responsible for relapse post-treatment, are resistant to many of the conventional therapies (Prbavathy et al., 2018). Similarly, the existence of a small population of breast CSCs is well proven, and these cells are thought to contribute to metastatic lesions. Breast CSCs cycle slowly, and there is accumulating evidence that they are the leading cause of resistance to conventional treatments. Various intracellular events govern CSC drug resistance and miRNAs have been proposed as playing a central role in governing the behavior of breast CSCs (Song and Farzaneh, 2012).

## How might we eliminate cancer stem cells?

The identification of LSCs, and subsequently of CSCs, has steered investigators towards searching for the means to eliminate these cells. The anticipated benefits would be the prevention of disease relapse and metastasis, and perhaps the provision of a bono fide cure for aggressive cancers that are hard to treat. Eliminating CSCs is a formidable and ongoing challenge because there is the need to spare normal stem cells as much as possible. As a starting point, targeting signaling events that play a crucial and selective role in governing the behavior of stem cells would spare their maturing and mature offspring. Activation of the RARs by all-trans retinoic acid (ATRA), the active metabolite of vitamin A, is well known to play a central role in controlling organogenesis during early embryonic development. The three main types of RAR, namely RARα, RARβ, and RARγ, form dimers with the retinoic acid X receptor. ATRA binding leads to the release of co-repressors of gene expression, the binding of co-activators, and gene expression.

Importantly, RARγ is selectively expressed by HSCs and their immediate offspring, and ATRA activation supports HSC self-renewal and maintenance as seen from a reduction in the number of HSCs in RARγ knockout mice (Purton et al., 2006). In contrast, it is well known that activation of RARα drives the differentiation of myeloid progenitor cells and promyeloid cell lines. From studies of early zebrafish embryos, RARγ expression is also restricted to primitive cells. They include mesodermal and neural crest stem/progenitor cells, in the head area, in the lateral plate mesoderm, and in the presomitic mesoderm of the tail bud (Hale et al., 2006). Treatment of embryos with a RARγ agonist blocked stem cell differentiation, preventing the formation of bone in the head area, neural ganglia, and fins. Tbx-5-immunopositive cells, for pectoral fin growth, were present in the appropriate region, and reversal of the action of the RARγ agonist, by the use of a RARγ antagonist or washout, restored fine growth (Wai et al., 2015). Taken together, the studies of hematopoiesis and zebrafish have shown that the activity statuses of RARγ and RARα mediate a balance between stem cell self-renewal and differentiation, playing opposing roles to promote stem cell maintenance and proliferation versus differentiation, respectively.

Hence, RARγ provides a putative target for eliminating CSCs, but further consideration is whether CSCs are more dependent than normal stem cells on the activation status of RARγ for their survival and/or proliferation. Activation of RARγ is vital to the survival and proliferation of prostate cancer (PCa) cells for three reasons. First, ATRA is at the limit of detection (∼1 ng/g tissue) within patients’ PCa tissue; adjacent normal cells and benign hyperplasia cells see ∼8 times more (Pasquali et al., 1996). PCa cells appear, therefore, to be adapted to survive and grow in an exceedingly low ATRA environment. Secondly, PCa cells and normal prostate epithelium express RARα and RARγ. For LNCap PCa cells, a sub nM level of ATRA (0.24 nM) transactivates RARγ, whereas 19.3 nM is needed to activate RARα (Brown et al., 2017). PCa cells appear, therefore, to be dependent on active RARγ for survival and/or proliferation. Finally, to test this assumption, the DU145, LNCaP, and PC3 cell line cells were treated with 10^–10^ to 10^–9^ M ATRA, to activate just RARγ, or a RARγ agonist; this enhanced cell survival and proliferation (Petrie et al., 2020).

In keeping with active RARγ plays a role in PCa survival is that antagonism of RARγ and all RARs eliminated the CSC-like cells that formed colonies when the DU145, LNCaP, and PC3 PCa cell line cells were plated into dishes (Petrie et al., 2020). The RARγ and pan-RAR antagonists killed the PCa cell line cells and primary PCa patients’ cells when grown in flasks. Normal prostate epithelial cells were 50% less sensitive than PCa cells to both of the antagonists, and blood mononuclear cells and fibroblasts were insensitive to the pan-RAR antagonist. It is important to note that the RARγ antagonist killed PCa CSC-like cells and all of their offspring and all of the cells within cultures of primary patients’ cells (Keedwell et al., 2004; Petrie et al., 2020). Therefore, there seems to be a failure to switch off RARγ expression when PCa CSCs mature to give rise to the population of overt cancer cells. As above, activation of RARγ promotes cell expansion, and in this way sustained RARγ expression within the offspring of CSCs might contribute to disease burden to adversely affect patient prognosis. Further support for the use of a RARγ antagonist to treat PCa is that a RARγ network governs androgen signaling within and disease progression of PCa, as several of the target genes (e.g., *SOX15*) are associated with worse disease-free survival (Long et al., 2019). Antagonism of RARs was effective against other cancers. Antagonizing all RARs killed MCF7 and MDA-MB-231 breast cancer cell line cells and pediatric patients’ primitive neuroectodermal tumor and astrocytoma cells, including elimination of the CSCs that gave rise to neurospheres (Brown and Petrie, 2021).

The three well-described modes of cell death are apoptosis (known as type I), autophagic (type II), and necrosis (type III). When Jurkat T-cell leukemia cells were deprived of ATRA they died *via* a caspase-independent and, therefore, non-apoptotic, process. They died *via* necroptosis which is a programmed form of necrosis involving the activation of poly-(ADP-ribose) polymerase 1 (PARP1) (Chiu et al., 2008). In turn, this has been associated with mitochondrial dysfunction and the release of ATP, NAD^+^, and the caspase-independent nucleases AIF and endonuclease G. RARγ antagonist treated PCa cells growth arrested in G1 of cell cycle and cell death was mitochondria depolarisation-dependent and cellular DNA fragmentation occurred that was caspase-independent (Keedwell et al., 2004; Petrie et al., 2020). PARP1 inhibition, by 1,5-dihydroisoquinoline, blocked the RARγ antagonist’s action, and, therefore, the antagonist-mediated cell death is *via* necroptosis.

The cytosolic death complex ripoptosome drives necroptosis. It comprises caspase 8, the kinases receptor-interacting protein 1 (RIPK1) and RIPK3 and the adaptor proteins FAS-associated DEATH domain (FADD) and TNFR1-associated DEATH domain (TRADD) (Wrighton, 2011). Cytosolic RARγ plays a pivotal role in driving necroptosis that is provoked by DNA-damaging compounds which led to RARγ being released from the nucleus to the cytosol. Loss of RARγ abolished DNA damage-induced necroptosis, as seen from studies of knockout mice and primary squamous cell carcinoma cells. These studies showed that RARγ is essential to RIPK1 and RIPK3 forming a complex, and the recruitment of the mixed lineage kinase domain-like protein (pMLKL) which is activated by RIP3 (Kadigamuwa et al., 2019). Cytosolic RARγ has also been shown to be pivotal to tumor necrosis factor (TNF)-induced cell death of HT-29 colorectal adenocarcinoma cells (Xu et al., 2017). Caspase 8, a component of the ripoptosome, is negatively regulated by the cellular inhibitor of apoptosis 1 (cIAP1) and cIAP2. For TNF-driven necroptosis of HT-29 cells, it has been shown that RARγ controls RIPK1-initiated cell death when the activity of cIAP was inhibited. RARγ mediated dissociation of RIPK1 from TNF receptor 1 to facilitate necroptosis. Antagonism of RARγ within PCa cells might, therefore, have led to RARγ accumulating in the cytoplasm.

Further support to targeting RARγ to eliminate CSCs is that RARγ is an oncogene for some cancers. Some AML patients’ cells harbour RARγ fusion proteins (Conserva et al., 2019). RARγ is often over expressed in colorectal carcinoma, and knockdown of *RARG* within the colorectal carcinoma cell lines HT29, HCT116, RK0 and SW480 enhanced their sensitivity to the chemotherapeutic drugs 5-fluorouracil, oxaliplatin and vincristine sulfate (Huang et al., 2017). RARγ overexpression in the chemo-resistant bile duct carcinoma cholangiocarcinoma is associated with a poor prognosis and resistance to 5-flurouracil (Huang et al., 2013). Overexpression of RARγ promotes the growth of hepatocellular carcinoma xenografts in mice (Yan et al., 2010) and occurs in around 50% of cases of clear cell renal carcinoma (Kudryavtseva et al., 2016). For gastric cancer, RARγ has been identified as an important regulatory TF from mRNA microarray studies of differentially expressed genes (Wang, 2017). RARγ overexpression within colorectal carcinoma, cholangiocarcinoma, and clear cell renal and hepatocellular carcinomas were measured for the cancer cell populations as a whole, rather than just for the CSC component. As above for PCa, the bulk of the above carcinoma cells seem to express RARγ and active RARγ maintains a stem cell phenotype. The presence of immature cells is a feature of cholangiocarcinoma and hepatocellular carcinoma. Progenitor cells give rise to these carcinomas with markers of progenitor cells. A very different view is that hepatocytes are the cell-of-origin with the transformed cells dedifferentiating into cells that express progenitor cell markers for hepatocellular carcinoma or transdifferentiating into bile duct-like cells for cholangiocarcinoma (Sia et al., 2017). Aside from this uncertainty, an inappropriate expression of RARγ may underlie a precursor cell phenotype.

There are efforts to develop immunotherapeutic approaches to eradicating CSCs. Chimeric antigen receptor T-cells (CAR T) have engineered synthetic receptors that direct these cells to recognize cells that express the target antigen at their surface. The aim is to target molecules that are selectively expressed by cancer cells. For B-ALL, the target antigens used for CAR T design have included, for example, CD19 and CD20, whereby CD19 is expressed throughout B-cell development and CD20 is a B-cell-specific antigen. CD20-based CAR T therapy, alone or in combination with CD19-based therapy, was highly efficacious in animal models of B-ALL (Riaz et al., 2017). Accordingly, CAR T therapy has led to effective and durable clinical responses for hematological malignancies, providing effective and safe treatment for ALL in phase I/II clinical trials (Sheykhasan et al., 2022). Even so, successes from the use of CAR T-cells are still rare and limited for solid tumors by the physical barrier of the niche the cancer cells reside in (Liu et al., 2019). A challenge to preclinical studies is that there is a lack of translational animal models that are relevant. Antibodies that target a cell surface antigen also provide a route to immunotherapy of CSCs, and, for example, there are endeavors to target epidermal growth factor receptor and human epidermal growth factor receptor three in the case of triple-negative breast cancer (Rau et al., 2020). For both CAR T and antibody targeting, there is still often the need to identify a proper target, including even for patients with the same type of cancer, and moreover, antigen expression by cancer cells is generally heterogeneous.

The major intracellular signaling pathways within CSCs also provide avenues that are being explored to target CSCs. Their importance to the control of the proliferation, apoptosis, and metastasis of CSCs and endeavors to target are reviewed extensively elsewhere (Chen et al., 2013; Yang et al., 2020). In brief, the signaling pathways include Wnt, NF-κB, Notch, Hh, JAK-STAT, PI3K/AKT/mTOR, TGF/Smad, and PPAR. For example, Wnt signaling has a role in regulating the differentiation status of CSCs and apoptosis of these cells, and studies of PI3K/AKT/mTOR signaling within cancer cells have revealed roles in the maintenance of stemness and cell survival. Some of the proposed strategies to targeting signaling pathways kill CSCs. Sulphoraphane and curcumin inhibit the NF-κB pathway and regulate the self-renewal of breast cancer CSCs and the apoptosis of hepatocellular carcinoma CSCs, respectively. Disulfiram, which is used to treat alcoholism, inhibits the metastasis of breast cancer CSCs via interfering with NF-κB/ERK/Snail pathway signalling. The adenosine 5′-monophosphate-activated protein kinase signaling pathway (AMPK) is important to glucose balance within CSCs. The antidiabetic drug metformin targets AMPK signalling and inhibits the proliferation of colorectal and hepatocellular carcinoma CSCs. Small molecule antagonists of Hh signalling that inhibit tumor cell growth have been described, such as GANT-61 (Lauth et al., 2007). Other efforts to target signaling pathways within CSCs are still under preclinical evaluation. And, it is important to note that there is cross-talk between pathways leading to the promotion of CSC maintenance and expansion, and, in turn, metastasis. A hurdle to the effective elimination of CSCs is the need to identify novel signalling within CSCs because they share many signaling pathways with normal stem cells.

In 2012, ferroptosis, a type of autophagy-dependent cell death, was described for *NRAS*-mutant HT-1080 fibrosarcoma cells treated with RAS-selective lethal compounds (e.g. erastin) (Dixon et al., 2012). It involves the iron-dependent accumulation of lipid hydroperoxides and is an emerging focus regarding the selective targeting of CSCs (Elgendy et al., 2020; Wu et al., 2021). In particular, there is growing evidence to support the view that oncogenic signaling renders cells susceptible to ferroptosis (Angeli et al., 2019). Medicinal chemistry has long been a dominant approach to the development of anticancer drugs, and investigators discovered small molecules with the potential to selectively kill CSCs when they developed new open-chain epothilone analogues. They killed mesenchymal breast cancer cells with CSC properties by harnessing reactive oxygen species to induce ferroptosis (Taylor et al., 2019).

Eliminating CSC may provide a cure for disease relapse. The frequency of CSCs within a tumor varies and does this affect the risk of relapse? For adenocarcinoma and squamous cell carcinoma of the lung, an observational prospective cohort study assessed the influence of the frequency of CSCs on relapse. CSCs were present at around three percent within primary cell cultures. Primary analyses showed that the risk of relapse was not affected by the frequency of CSCs. For patients with locally advanced disease, the risk of relapse was increased by a higher frequency of CSCs (Masciale et al., 2022). The findings for other tumors will be of interest regarding whether the frequency of CSCs is an important consideration to relapse. Other factors are important. Longitudinal analysis of cell-free DNA from patients with small cell lung cancer has brought to attention the importance of treatment efficacy and the frequencies of mutant alleles and copy number alterations. Disease-associated mutations were observed in eight-five percent of patient samples and mutant allele frequencies ranged from very low (0.1%) to eighty-seven percent. The response to treatment tracked closely with mutant allele frequencies and alterations to copy number (Almodovar et al., 2018). For breast cancer, the recurrence of metastatic disease is varied and unpredictable with investigators commenting that there are “*lingering mysteries of disease recurrence*” (Riggio et al., 2021).

## Perspectives and conclusions

Advancing knowledge about the different types of leukemias coupled to an understanding of the architecture of the hematopoietic cell system brought leukemia to the forefront as a model system for cancer. The relative ease of accessibility of patients’ cells contributed, and, from the 1980s, hematopoietic cell lines were generated that typified almost all of the subsets of the lymphoid and myeloid leukemias (except for CML chronic phase) (Drexler et al., 1999). It might be argued that leukemia is an inappropriate mode for cancer because metastasis of prevalent carcinomas is a longstanding and major challenge to treating cancer successfully. However, leukemia is not exceptional because, from studies of the anatomy of the leukemias, it has been argued that their dissemination is a metastatic process (Whiteley et al., 2012).

Findings from studies of AML markedly shaped our view of the nature of cancer. They were that a rare subpopulation of cells, namely LSCs, sustains the hierarchy of leukemia cells and that LSCs share unique biological properties with HSCs. An attractive hypothesis, from consideration of the cell of origin of some leukemias, is that many cancers may arise directly from a tissue-specific stem cell. Seminal studies of B-ALL have shown that, at least, two genetic insults are required for leukemia. Some leukemias arise from an HSC and there is still the need to unravel how each of the insults contributes to an alteration to cell behavior. The targeting of some oncogenes to HSCs/HPCs restricted LSCs to one differentiation pathway. Mathematical modeling has revealed a high order of multi-stability for HSCs, rather than bi-stability, with noise and bursting of gene expression playing a role in HSCs veering towards a lineage (Wou et al., 2022). This plasticity is essential to the stem cell state, and HSCs may shift towards a lineage option by guidance from the environment. The hematopoietic cytokines erythropoietin and macrophage colony-stimulating factors guide erythroid and myeloid fate within HSCs, respectively (Mossadegh-Keller et al., 2013; Grover et al., 2014). As to the bursting of gene expression, the transformation of an HSC by some oncogenes may enforce a lineage affiliation at a moment in time of a particular burst of gene expression.

Conventional treatments that are often highly effective against the bulk of cancer cells spare LSCs/CSCs. Agents that drive apoptosis are less effective against CSCs because many cancers are intrinsically resistant or acquire resistance to the induction of apoptosis (Fulda, 2009), and necroptosis is considered as a “fail-safe” death pathway for apoptosis-resistant cells (Gong et al., 2019). Pan-RAR and RARγ antagonists were potent inducers of necroptosis of PCa, breast cancer, and pediatric patients’ brain tumor CSCs and their offspring, and normal prostate epithelium cells were less sensitive than PCa cells. A number of other compounds are known to drive necroptosis of cancer cells, the list is growing, and it includes compounds that drive necroptosis of apoptosis-resistant cancer cells (Fulda, 2013).

The stem cell theory of cancer is still a work in progress. Though CSCs have been identified for a good number of human solid tumors, whether the risk of relapse is increased by a higher frequency of CSCs needs further investigation for patients at presentation and with locally advanced disease. As for all cancer treatments, the aim is to kill or control cancer cells avoiding damage to normal tissues. This issue is highly pertinent to endeavors to kill CSCs when the normal counterpart cell is a tissue-specific stem cell because of its importance to replenishing worn out cells. These two cell populations share many characteristics, as seen for signaling pathways. Finding specific attributes of CSCs, for targeting, requires a much clearer picture of their nature versus their counterpart tissue-specific stem cell. Whether most, if not all, malignancies arise directly from a tissue-specific stem cell is still debatable. If so, how the progeny of LSCs/CSCs become restricted to a cell lineage is, as yet, unclear. The resistance of many cancer to apoptosis has prompted the need to explore driving other cell death modalities, such as necroptosis or ferroptosis. In this case, whether CSCs are more sensitive than normal tissue-specific stems cells to the induction of these death modalities is an important issue. The search is for agents that kill CSCs and that spare tissue-specific stem cell populations. The resolve to this problem may well require the development of new synthetic compounds that target the specific attributes of CSCs when we have a clearer picture of their nature.
